# Belladonna in Pertussis

**Published:** 1834

**Authors:** 


11. *Belladonna in Pertussis.*—In the *Amer. Journ.* Dr. Jackson of Northumberland, has published the results of some recent trials with the extract of Belladonna, in hooping cough. They are highly encouraging. This powerful antispasmodic, repeatedly given in pertussis by the physicians of Germany, has been but seldom employed in the United States. We hope the experience of so respectable a practitioner, as the gentleman whom we are about to quote, will incite others to the use of this medicine, in one of the most refractory of the diseases to which our children are liable. The following course of preparation will in general be required.

"If any one then shall condescend to try this method of curing hooping-cough, let him first remove the greater part of the inflammation by bleeding, laxatives, low diet, nauseating doses of tartar emetic, and an abundance of milk-warm linseed tea. Bleeding and blistering may be necessary at the onset, and even in the progress, if inflammation supervene as it is very apt to do upon weak or unsound lungs. Leeching or cupping may often be more suitable, nor can it require much medical acumen to determine on this point. But many cases will occur in which the belladonna may be given almost from the first, at the same time that the above-mentioned antiphlogistics are in use."

The extract of belladonna is often adulterated or effete. Its activity may however be easily tested, by dropping a little of the solution into the eye, or even rubbing it over the lids, as the well-known dilatation of the pupil will ensue, if the medicine be good. We should remark, however, that this dilatation is much earlier, wider, and of longer continuance, in some eyes than others.

"Suppose then, that the system is well prepared for the belladonna, and that you have some which is certainly of good quality; let the extract be triturated with water, to which, in order to prevent fermentation, add a suitable proportion of proof spirit; give this to a child three months old in such doses every three hours, from sunrise till bed-time; that each dose shall contain one-sixth of a grain of the extract; to a child of two years old a full grain may be given; to one four years a grain and a half, and from these data the medium dose for any age can be easily ascertained. But with respect to the dose, it is certain that we know but little, as our experience has been very limited, and the strength of extracts is very uncertain.

The effects on the system must be carefully watched, and as soon as dilatation of the pupil takes place, the medicine must either be omitted altogether, or given in much smaller doses. We consider it as far the safest to give no more until we may be satisfied that the effects of the first exhibition are beginning to pass off. The reason of this is too evident to the regular practitioner, and others must take the fact, if they are not capable of the reasoning.

As soon as the system begins to rise from under its influence, which may be known in the infant by the state of the pupil, let it be resumed, and given in larger doses than before, so as to make a strong impression. It is now that its powers become apparent, but the dose must be increased, for, like conium, its effects on the system diminish most wonderfully in proportion to the doses administered. The disease will now begin to yield, and how much longer the medicine is to be continued, or what coädjuvants may or ought to be used, let every judicious physician determine for himself, when the case with all its necessities are before him. We are not writing a treatise on the subject of pertussis, but merely endeavoring to show the great and almost incredible power of a single medicine therein.

